# Role of magnesium ions in the reaction mechanism at the interface between Tm1631 protein and its DNA ligand

**DOI:** 10.1186/s13065-016-0188-6

**Published:** 2016-07-08

**Authors:** Mitja Ogrizek, Janez Konc, Urban Bren, Milan Hodošček, Dušanka Janežič

**Affiliations:** National Institute of Chemistry, Hajdrihova 19, 1000 Ljubljana, Slovenia; Laboratory for Physical Chemistry and Thermodynamics, Faculty of Chemistry and Chemical Technology, University of Maribor, Smetanova ulica 17, 2000 Maribor, Slovenia; Faculty of Mathematics, Natural Sciences and Information Technologies, University of Primorska, Glagoljaška 8, 6000 Koper, Slovenia

**Keywords:** DUF72, Tm1631, QM/MM, Unknown function, ProBiS, CHARMM, GAMESS, *Thermotoga maritima*

## Abstract

A protein, Tm1631 from the hyperthermophilic organism *Thermotoga maritima* belongs to a domain of unknown function protein family. It was predicted that Tm1631 binds with the DNA and that the Tm1631–DNA complex is an endonuclease repair system with a DNA repair function (Konc et al. PLoS Comput Biol 9(11): e1003341, 2013). We observed that the severely bent, strained DNA binds to the protein for the entire 90 ns of classical molecular dynamics (MD) performed; we could observe no significant changes in the most distorted region of the DNA, where the cleavage of phosphodiester bond occurs. In this article, we modeled the reaction mechanism at the interface between Tm1631 and its proposed ligand, the DNA molecule, focusing on cleavage of the phosphodiester bond. After addition of two Mg^2+^ ions to the reaction center and extension of classical MD by 50 ns (totaling 140 ns), the DNA ligand stayed bolted to the protein. Results from density functional theory quantum mechanics/molecular mechanics (QM/MM) calculations suggest that the reaction is analogous to known endonuclease mechanisms: an enzyme reaction mechanism with two Mg^2+^ ions in the reaction center and a pentacovalent intermediate. The minimum energy pathway profile shows that the phosphodiester bond cleavage step of the reaction is kinetically controlled and not thermodynamically because of a lack of any energy barrier above the accuracy of the energy profile calculation. The role of ions is shown by comparing the results with the reaction mechanisms in the absence of the Mg^2+^ ions where there is a significantly higher reaction barrier than in the presence of the Mg^2+^ ions.

**Electronic supplementary material:**

The online version of this article (doi:10.1186/s13065-016-0188-6) contains supplementary material, which is available to authorized users.

**Graphical abstract:**
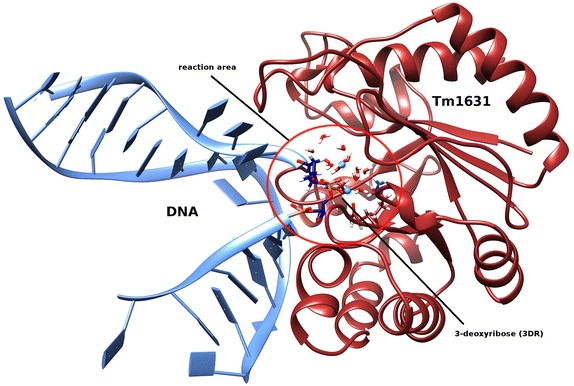
A protein, Tm1631 from the hyperthermophilic organism Thermotoga maritima belongs to a domain of unknown function protein family. We modeled the reaction mechanism at the interface between Tm1631 and its proposed ligand, the DNA molecule, focusing on cleavage of the phosphodiester bond

## Background

Tm1631 is a member of the domain of unknown function 72 (DUF) family in the Protein family (Pfam) database, a protein domain that has no characterized function [1]. Protein function can only be unambiguously determined experimentally, but in case of a new protein with no computationally predicted putative function it is difficult to choose the correct experiment. New procedures are necessary to facilitate this research and improve determination of function for all the DUF proteins. A new approach to this problem has been developed by our group and was described in a previous report to predict the function of the Tm1631 protein [2].

**Fig. 1:**
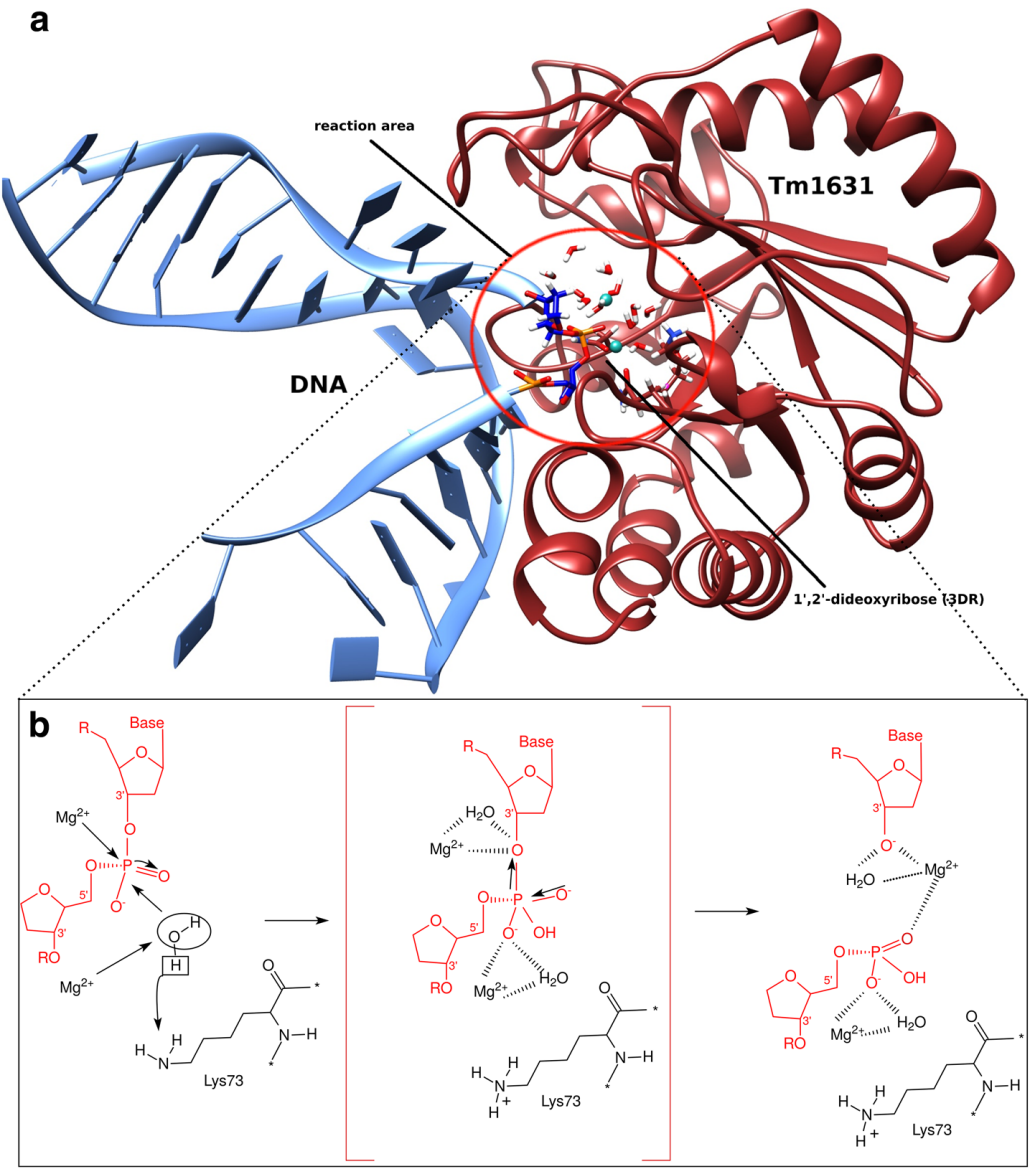
The proposed reaction mechanism for Tm1631–DNA complex. **a** Reaction area is *encircled*. The damaged nucleotide abasic dideoxyribose (3DR), which has no base (abasic site), is on position 7 of the 15 base pair long DNA chain. **b** Structure-based reaction mechanism of phosphodiester bond cleavage. The abasic site on the DNA is coordinated by the two Mg^2+^ ions, of which one also attacks the hydroxyl nucleophile (*left panel*). Pentacovalent transition state [20–22] (*middle panel*) collapses, which leads to the cleavage of the scissile phosphodiester P-O3′ bond, with the transition state and the O3′ leaving group stabilized by the metal ion and inversion of the phosphate configuration (*right panel*)

In an earlier paper [2], we used the binding site comparison capability of the ProBiS algorithm [3, 4] to predict the binding site in the Tm1631 protein and to speculate on the nature of the DNA ligand that could bind to the Tm1631 protein. The Tm1631 protein was predicted to be analogous to endonuclease IV despite sharing <10 % sequence identity, and the proposed Tm1631–DNA complex was subjected to 90 ns long classical MD, using the CHARMM simulation package [5]. This resulted in structures of the Tm1631–DNA complex that are used in the QM/MM study reported in this paper, where we attempted to investigate the catalytic mechanism of the reaction between Tm1631 and DNA. It was of especial interest in this connection to determine how Mg^2+^ ions act in this DNA binding site (Fig. 1). Endonuclease repair mechanism is an important mechanism that allows organisms to escape DNA damage and plays a major role in the prevention of cancer in higher organisms [6]. Endonucleases cleave phosphodiester bond of DNA at the damaged site and create a nick in the phosphodiester backbone that is recognized by further repair enzymes in the base excision repair pathway. The endonuclease catalytic mechanism is thought to involve a hydroxide ion derived from water, which forms a bond with phosphorus of the DNA and induces cleavage of the phosphodiester bond.

Endonucleases require one, two or three divalent metal ions, such as Mn^2+^ or Mg^2+^ in the catalytic site [7–13]. It is believed that in thermal environment, such as the one *Thermotoga maritima* lives in at temperatures around 80 °C, the most suitable metal ion for this kind of system is Mg^2+^ [14]. However, Tm1631 crystal structure (PDB: 1VPQ) does not contain any metal ions in the predicted binding site. On the other hand, there are relatively few crystallographically characterised magnesium binding sites [15]. For some binding sites, a metal ion is observed in the binding site but which metal ion it is and how many of them are needed for the catalytic activity remains undetermined [16–19].

Here, we report the results of theoretical QM/MM studies of the reaction mechanism for the Tm1631 protein. We postulated a catalytic mechanism with two Mg^2+^ ions that resembles the one of the apurinic/apyrimidinic (AP) endonuclease enzyme presented by Mol et al. [21]. We tested the system with and without the ions, and found that the energetically most favourable pathway of the phosphodiester bond cleavage catalysed by Tm1631 requires presence of Mg^2+^ ions. In the proposed catalytic mechanism (Fig. 1b), Lys73 of the Tm1631 should be in a deprotonated state before water ionizes. One of the Mg^2+^ ions attacks the water molecule, and subsequently water ionizes, the proton forms a bond with Lys73 and the OH group forms a bond with the phosphorus atom. The transition state then collapses leading to cleavage of the phosphodiester P-O3′ bond, while the O3′ leaving group is stabilized by the second Mg^2+^ ion. Our findings suggest that the catalytic mechanism of Tm1631 requires Mg^2+^ ions and is similar to known Steitz’s mechanism [23].

Experimental and computational studies [24, 25] point out that the cleavage of the phosphodiester bond occurs via S_N_2 nucleophilic substitution in three steps (Fig. 1b) explained in work by Sgrignani et al. [26] and Yang et al. [27].

## Methods

Calculations were based on the crystal structure of protein Tm1631, (PDB: 1VPQ) and the DNA chains from another crystal structure (PDB: 2NQJ).

### System setup and MD

The structures used in this work were previously equilibrated by classical MD simulation. Since there is no crystal structure of Tm1631 with DNA available in the Protein Data Bank (PDB), we used as the starting structure for this study the predicted Tm1631–DNA complex after 90 ns of classical MD, which was performed in our previous study [2]. To validate this starting complex structure, we plotted its all-atom, protein Tm1631 and DNA root-mean-square deviations (RMSDs) (compared to the first snapshot at 0 ns of classical MD) dependence against the simulation time, which showed that in the last 20 ns of simulation the RMSDs have reached a plateau, suggesting that the starting structure is well equilibrated (Additional file 1: Figure S1).

In order to obtain good reactant and product structures we probed different Mg^2+^ ion and water molecules positions. In this search we required that Mg^2+^ initially coordinates with six oxygen atoms (Additional file 1: Table S1; Distances to Mg_1_^2+^ and Mg_2_^2+^), and with the distant environment atoms in the same positions for both, the reactant and product conformations. The final energy minimization procedures were performed without any constraints or restraints and we were able to obtain suitable starting positions for the reaction mechanism studies with the above mentioned properties.

### Model building and QM/MM simulation

**Fig. 2:**
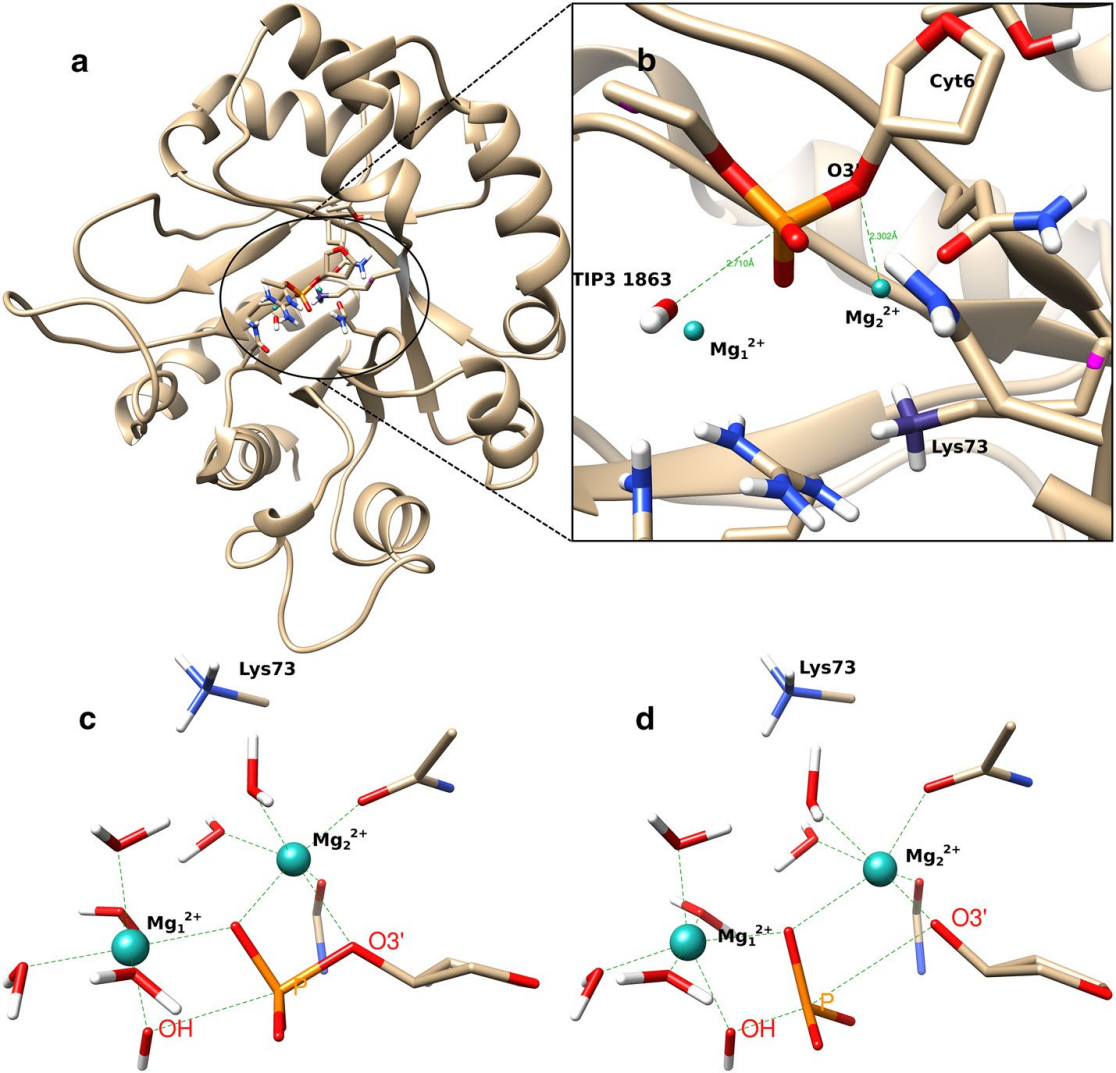
Protein Tm1631, Mg^2+^ ions, and important amino acids in the QM region. **a** Protein Tm1631 and **b** zoom in of the binding pocket used for QM/MM calculation. Protein residues that are considered as QM and 3DR7 residue of the DNA are denoted as stick models, and the two Mg^2+^ ions are *cyan spheres*. One of four link atoms is *pink*, others are not visible. In **c** reactant and **d** product are coordinated with both Mg^2+^ ions (*cyan spheres*), each being coordinated with 6 oxygen atoms in total. The distance between O3′ and Mg_2_^2+^ decreases from reactant (2.302 Å) to product (1.841 Å)

The starting structure of our simulation was the Tm1631–DNA complex after 90 ns of classical MD simulation. We replaced two water molecules with two Mg^2+^ ions and then minimized the system. A minimized structure with Mg^2+^ ions was further optimized at quantum mechanics/molecular mechanics (QM/MM) level using the CHARMM software package. The CHARMM force field parameters were used to describe the molecular mechanics (MM) part, while the quantum mechanics (QM) region (42 atoms in total, including both Mg^2+^ ions (Fig. 2a, b) was treated at the density functional theory (DFT) level using the B3LYP functional and the 6–31G* basis set. We used the Replica path (RPATh) method to divide the system into 16 structures equidistantly apart in the RMSD space between the reactants and products and minimized each obtained structure using 3000 steps of adopted basis Newton–Raphson (ABNR) minimization. After minimization we checked the distances between Mg_1_^2+^ and Mg_2_^2+^ ions, which were both around 4 Å. Both Mg^2+^ ions were coordinated with 6 oxygen atoms (reactants, Fig. 2c; product, Fig. 2d), and the distances between coordinated atoms and both Mg^2+^ ions were approximately 2 Å.

### QM/MM

Ab initio QM/MM [28] methods were performed with the CHARMM biomolecular program [5]. In the molecular mechanics part of the calculations, we used CHARMM force field version 36. The ab initio DFT calculations were performed using the general atomic and molecular electronic structure system (GAMESS) [29] software package, interfaced to the CHARMM program. We used the B3LYP/6–31G* level of theory, which is implemented in the GAMESS program. The ABNR minimization algorithm was used for energy minimizations. Molecular mechanics calculations were performed with a constant dielectric of ε = 1 using a classical force shift method and a cut-off distance of 12 Å. Molecular graphic images were produced using the VMD software package [30]. All ab initio QM/MM and QM calculations were carried out on the CROW clusters at the National Institute of Chemistry in Ljubljana [31].

### RPATh

Chain-of-replica methods involve discretising any reaction pathway by defining replicated conformations along the path between the reactants and products [32]. In order to keep the pathway along the reaction smooth a penalty term is included into the potential function which keeps the RMSD values equidistant between all the neighbouring conformations [33]. This modified potential function is then used in the geometry minimization procedure to obtain the minimum energy pathway between the reactant and product structures. Since the penalty is in the RMSD space there is no preference in the reaction coordinate to the individual distances among the atoms and is thus an efficient tool to investigate the order of bond breaking and bond making during the reaction process. The RPATh method can be used for both minimizations and the MD simulations. In order to investigate the basic steps of the reaction process, one first explores the potential energy surface by minimum energy pathway from reactants to products by a minimization procedure. In the RPATh setup this means that all the replicas are minimised along with the restraint which forces them to be equidistant in RMSD space. To completely understand the energetics of the reaction process one must calculate the free energy along the reaction pathways. Unfortunately, it is not possible to perform such calculations with the satisfactory accuracy using the currently available computational methods, although much effort is invested to develop practically feasible QM/MM methods to calculate the free energy of a reaction processes [34, 35].

### Study of concurrent reaction mechanisms

The following steps define the procedure for the reaction with two Mg^2+^ ions in the binding pocket:

1. For coordination and structural files, we read the last coordinate frame from the file produced by the 90 ns classical MD simulation as explained earlier [2].
2. To perform QM/MM calculations, the QM region and if necessary also the boundary between the classical potential and the quantum potential involving link atoms must be defined. We assigned the QM region as follows: Lys73 side chain, two water molecules close to reaction center: water number 1863 (2.710 Å; Fig. 2 and Additional file 1: Table S1) is involved in the reaction and nearby water 6134, phosphate group with adjacent C5′ atom from abasic dideoxyribose on place 7 in the DNA (residue 3DR7), and the 5-member ring from Cyt6 residue on the DNA. We used 4 link atoms, because we divided QM and MM region in the middle of four covalent bonds: one in Lys73, one in 3DR7, and two in Cyt6 residues. The total number of QM atoms in the system including two Mg^2+^ ions was 42 consisting of 170 electrons described by 343 basis functions. The total number of atoms in the system was 13,932, including 2860 water molecules.
3. A reactant was constructed for which the QM/MM minimization procedure was initiated by using RESD [36] restraints for bonded atoms P (3DR7) and O3′ (CYT6) so that they remained separated at 1.6 Å and for OH^−^ (water 1863) and P (3DR7) which remained 3.5 Å apart. On both bivalent metal ions, the Mg^2+^ harmonical restraint (the “cons harm” command in CHARMM) was used to preserve the coordination of ions. At this stage 100 steps of ABNR minimization were performed. Then we removed CONS HARM restraint from the minimization run of 300 steps with only RESD restraints for bonds left. Subsequently we removed all the restraints and ran 1500 steps of minimization.
4. From the structure of the reactant we made a product, breaking the bond between P (3DR7) and O3′(CYT6) and created a bond between OH^−^ (water 1863) and P (3DR7) 1.6 Å using restraints. We also restrained the distance between O5′ (3DR7) and P (3DR7) at 1.6 Å. After 300 steps with RESD, we removed all the restraints and ran 1500 steps of a geometry minimization procedure.
5. We replicated the whole system 16 times and produced initial replica conformations by linear interpolation of the coordinates between the reactant and the product. The first and last replica represented reactant and product conformations, respectively, and were fixed throughout the pathway minimizations. With this setup a 3000 steps ABNR minimization was performed.

Procedure steps for reaction without Mg^2+^ ions in the binding pocket:

1. We started with the minimized reactant from the simulation with two Mg^2+^ ions, then removed them from the structure and held the distance between OH^−^ (water 1863) and P (3DR7) at 4 Å for 300 steps. Afterwards we removed all RESD restraints and ran 3000 steps of ABNR minimization.
2. We took the product from the previous simulation with two Mg^2+^ ions and removed them; subsequently running 3000 steps of an ABNR minimization procedure.
3. We performed an RPATh calculation, with 3000 steps of ABNR minimization procedure using identical number of replicas as in the calculations with ions present in the system.

To check the influence of the protein environment on the lowering of the energy barrier, we performed pure QM calculations with the GAMESS program as well as the reaction path calculations with the RPATh method. B3LYP/6–31G* level of theory was also used for all calculations in the pure QM case.

Procedure steps for reaction without Mg^2+^ ions and pure QM calculations:

1. First we made the reactant state from the already QM/MM minimised structure without Mg^2+^ ions. Subsequently we removed the whole protein, DNA structure and water molecules that were not in the QM region. Finally we ran 3000 steps of ABNR geometry minimization with restrains, holding all three protons attached to the Lys73.
2. We made a product from the QM/MM calculations without Mg^2+^ ions in the binding pocket. Then we ran 3000 steps of ABNR minimization without restrains.
3. RPATh calculation was finally performed with 3000 steps of ABNR minimization, using 16 replicas.

## Results and discussion

The Tm1631 protein with yet unknown function from the organism *T. maritima* has a similar binding site to that of DNA repair proteins, as established earlier [2]. By exploring the possible reaction pathways using QM/MM methods we tried to gain insight into the catalytic mechanism of the Tm1631–DNA complex. The similarity of the energetically most favourable pathway of the Tm1631–DNA complex with that of Mol et al. [21] strongly suggests that the mechanism is the same as in other endonucleases. The mechanism for Mg^2+^ ions catalysis that we propose is most likely the so-called Steitz’s mechanism [23, 37].

A variety of conformations within the active site were energetically evaluated and compared and the following systems were studied:

1. QM/MM calculation for the reaction with 2 × Mg^2+^ ions in the binding pocket (Additional file 2: Video S1).
2. QM/MM calculation for the reaction without Mg^2+^ ions in the binding pocket.
3. QM calculation for the reaction without Mg^2+^ ions in the binding pocket and environment with no protein or solvent molecules.

Quantum mechanics/molecular mechanics and QM calculations were performed using the B3LYP/6–31G* DFT method. As expected, obtained results suggest that the protein has an impact on lowering the reaction barrier and also establish that metal ions are required in the binding pocket. Our interest in this study is focused on the core DNA repair function, leaving the deprotonation of Lys73 for future investigations, however there is evidence that such a state exists [8]. As explained in the previous section we made sure that the ions are coordinated as expected [38]. We are aware of the possibility that one, two or three ions may be involved in the DNA repair reaction mechanisms. However we present in this paper the results for the two ion systems and only the core part of the repair mechanism where the hydroxide ion attacks the phosphate and the P-O3′ bond gets cleaved.

The hydroxide ion involved in nucleophilic attack on the phosphodiester bond P-O3′ was derived from water, ionization of which is accomplished with the help of an Mg^2+^ ion. Then proton from the same water molecule forms a bond with the nitrogen atom of the side chain of the Lys73, which in its deprotonated state can act as proton acceptor in the enzyme’s active site [39], and is part of the nucleic acid repair mechanism [8]. We calculated the pKa of the Lys73 to be 9.55 using the DEPTH tool [40], which is the lowest of all lysine residues of the Tm1631 protein. We also calculated the average pKa of the Lys73′s surrounding residues (10.5), which suggested that Lys73 is most likely in a deprotonated state before reaction occurs and can accept a proton from water molecule (Additional file 1: Figure S2). Literature reports that Mg^2+^ plays a functional role in the catalytic mechanism and the stability of protein-DNA complex. Metal ions also lower the local pKa, and this, considering the harsh environment that the organism experiences, is in a good agreement with our study [7, 8, 41–43].

Magnesium ions coordination is essential for most phosphoryl transfer enzymes [44]. The common catalytic mechanism was proposed previously [23]. This mechanism works on the same principle as our proposed mechanism: Mg_1_^2+^ coordinates the nucleophile and Mg_2_^2+^ coordinates the leaving oxygen atom (O3^′^). Many similar systems with two Mg^2+^ ions in the binding pocket have been studied [12, 37]. Distance between both metal ions should be ~4 Å and in our case it is 3.85 Å (reactant) and 4.18 Å (product). Our system is also coordinated in the octahedral shape and most of the angles are 90° between O–Mg–O. Also seen from other enzymatic literature enzymatic phosphate hydrolysis proceeds as S_N_2-like nucleophilic attack on the scissile phosphate performed by an hydroxide ion, which is formed upon water activation [24–26, 45]. The important role of Mg^2+^ ions is lowering the pK_a_ of its ligands and also for the presence of second metal ion (which coordinates nucleophilic water or hydroxide in the binding pocket). This leads to a mechanism with early proton transfer [46, 47], preceding the cleavage of phosphodiester bond in case on RNase catalytic system. Mg^2+^ ions have an essential contribution for the specific catalytic reactions by lowering the pK_a_ of the leaving group and can impose specific geometry for the triphosphate chain—pentacovalent transition state and products [44].

**Fig. 3:**
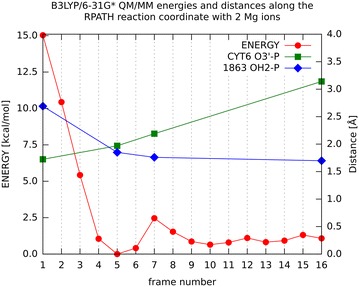
Energy profile (*red*) for each frame (1–16) of the QM/MM calculation with two Mg^2+^ ions; the values for energy are on the* y1 axis*. Frame numbers (1–16) represent steps on the reaction path, in which 1 is reactant and 16 is product. Values for distances between atoms Cyt6 03′—P (3DR7) (*green*) and water 1863 OH2-P (3DR7) (*blue*) are marked on the* y2 axis*

We observe six critical points in the energy profile for two Mg^2+^ ions shown in Fig. 3. The minimum with the lowest energy is the frame 5, which is very close to a symmetric structure, the so-called pentacovalent intermediate, with P atom in the center, three oxygen atoms (O1P, O2P, O5′) in a planar arrangement, and the two reacting oxygen atoms positioned almost equidistant on the opposite sides of the plane. Usually such a structure would suggest a transition state in the reaction pathway but the Mg^2+^ ions effectively stabilize the energy of pentacovalent intermediate to make it a stable minimum. In order to check this structure and its energy we performed a separate calculation. We minimized the geometry of the single number five replica structure. After 10,000 steps of ABNR minimization the geometry had no visible changes to the one in the chain of replicated structures and the energy was lowered by 2.5 kcal/mol due to the removal of the restraint which slightly distorts the distances and makes the energy higher. The effect of the distortion could be made smaller by increasing the number of replicas, however this would not change the overall properties of the reaction mechanism. At the current level of accuracy one can estimate that energy noise level in Fig. 3 is <3 kcal/mol. This makes the reaction kinetically controlled exothermic and not thermodynamically controlled because there are no energy barriers between the reactants and the products. Our calculated energy change (−14.0 kcal/mol) agrees with results of another study [26], in which energy difference of phosphodiester bong cleavage starting from OH^−^ was found to be −18.1 kcal/mol. The distances to important amino acids are reported in Additional file 1: Table S1.

**Fig. 4:**
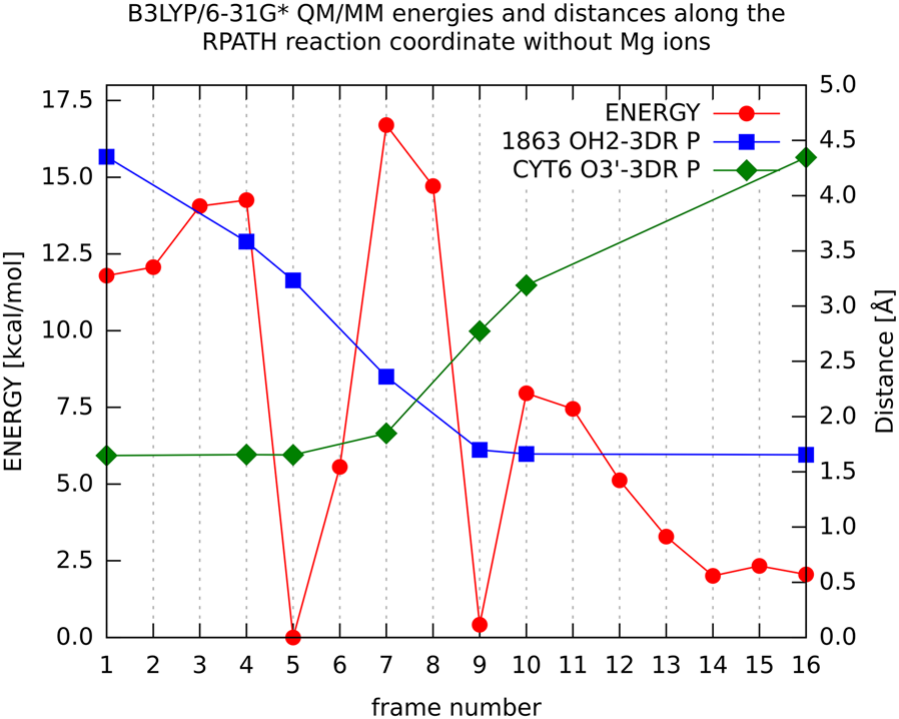
Energy profile (*red*) for the QM/MM calculation without ions with the values for the energy reported on* y1 axis*. Values for distances between atoms Cyt6 03′—P (3DR7) (*green*) and water 1863 OH2–P (3DR7) (*blue*) are marked on the* y2 axis*. The distances to important amino acids are collected in Additional file 1: Table S2

Next, we present the results of QM/MM calculations without Mg^2+^ ions (Fig. 4), which supports two observations:

- There is a high 17 kcal/mol barrier in the middle of the reaction path between structures five and nine. This structure has features analogous to those of structure six from Fig. 3, but in this case it is a transition state structure suggesting that the role of the Mg^2+^ ions is to transform this configuration into a stable and low energy pentacovalent intermediate.
- Many chemical reactions have multiple reaction channels which depend mostly on the positions of the species entering the reaction. In the case where the two Mg^2+^ ions are not present the conformational space of the entering species is larger than the one with the two ions present. This makes the reaction energetically less favourable because the system may explore more pathways which are of higher energy than the ones with the ions present. This works in addition to the fact that the environment atoms in the enzyme systems usually lower the energy barriers of any transition state structure. At this point we can add that a possible solution to choose the most favourable reaction channel would be the use of QM/MM molecular dynamics. But there is a sampling problem to be emphasized which, to be resolved, would require tens of nanoseconds of simulation time what translates into tens of millions steps of complete QM/MM calculations. In the present studies less than 100 thousands steps of QM/MM calculations were performed and still it took a few months of CPU time using between 32 and 128 processors depending on the task.

**Fig. 5:**
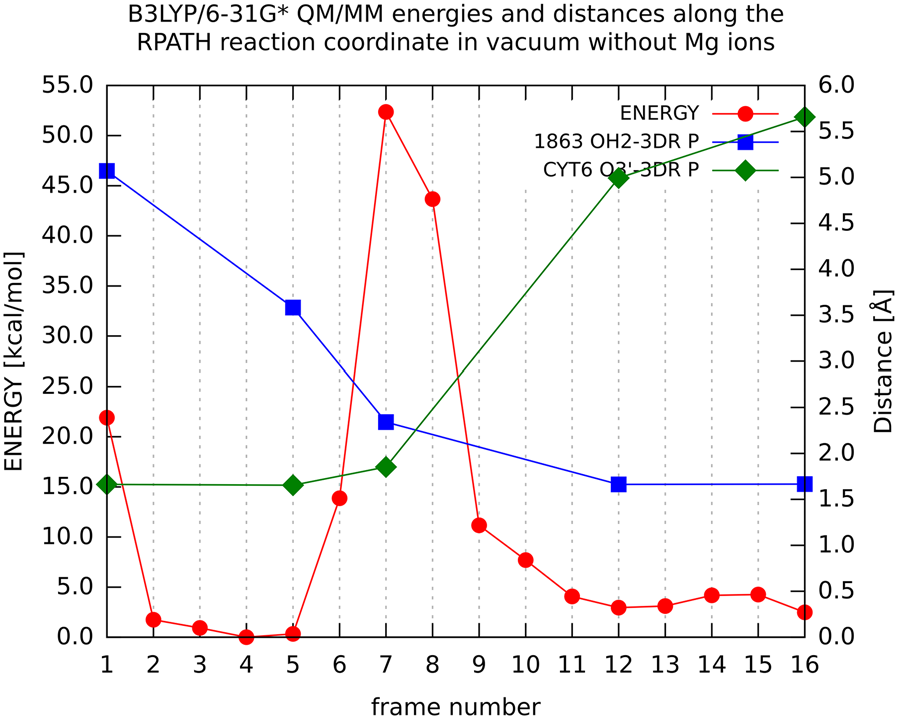
Energy profile (*red*) for QM/MM calculation in vacuum without Mg^2+^ ions with the values for the energy denoted on the* y1 axis*. Values for distances between atoms Cyt6 03′—P (3DR7) (*green*) and water 1863 OH2-P (3DR7) (*blue*) are marked on the* y2 axis*

To show the impact of the protein, DNA, solvent and ion environment on the studied reaction, we also studied the system in vacuum without Mg^2+^ ions. The vacuum calculation was set up keeping just the QM region in the reaction path calculations. From the Fig. 5 one can observe similar behaviour as in the QM/MM calculation without ions, but the barrier is extremely high which suggests that the protein environment indeed significantly contributes to the energy stabilisation of the pentacovalent transition state structure.

In order to verify the stability of the initial QM/MM setup we performed two additional classical MD 50 ns simulations starting with the reactant and product structures that we used for the minimum energy pathway calculations. The ions in these simulations kept the hexacoordinated structures throughout the simulations. In the case of reactant simulations none of the waters were exchanged and all 12 atoms around the two ions were identical. This means that the atom positions of reactant are in the stable and favorable positions to enter the reaction.

## Conclusion

Protein Tm1631 from the organism *T. maritima* was predicted to be an endonuclease-like DNA binding protein, and consequently we investigated its function focusing specifically on the role of Mg^2+^ ions in its binding pocket. We performed a QM/MM study of Tm1631 in a complex with damaged DNA. We found that Mg^2+^ ions are required in the binding pocket in order that the reaction occurs. This allows us to conclude that Tm1631 is indeed an endonuclease binding protein with a reaction mechanism similar to that of other endonucleases. Some reconciliation is still needed regarding the number of metal ions, e.g. it is possible that only one ion suffices for the reaction to take place. The present paper is one of the few theoretical insights in the available literature to study a series of the reactions that play a role in the complex of the endonuclease repair process. Future work should be aimed at determination of the precise number and type of ions that are needed for the reaction to occur. Another interesting study would be to explain the formation of the hydroxide ion in connection with the protonation of Lys73 and the role of ions in such mechanisms. It would also be interesting to compare the present results with the results obtained by similar studies in different proteins (Additional file 3).

## Additional files

10.1186/s13065-016-0188-6 Table of distances between important atoms; RMSD graph; pKa graph.
10.1186/s13065-016-0188-6 Movie of reaction mechanism.
10.1186/s13065-016-0188-6 PDB structures for all studied mechanisms.
