# Supplementary material for: Role of magnesium ions in the reaction mechanism at the interface between Tm1631 protein and its DNA ligand
Source: Chem Cent J. 2016 Jul 8;10:41. doi: 10.1186/s13065-016-0188-6 (PMC4939058; doi:10.1186/s13065-016-0188-6)
Supplement: Supplementary file 1 — 10.1186/s13065-016-0188-6 Table of distances between important atoms; RMSD graph; pKa graph. [file 13065_2016_188_MOESM1_ESM.doc]

**SUPPORTING INFORMATION**

Role of magnesium ions in the reaction mechanism at the interface between Tm1631 protein and its DNA ligand

Mitja Ogrizek^1^, Janez Konc^1,2,3^, Urban Bren^1,2,3^, Milan Hodošček^1,*^, Dušanka Janežič^3,*^

^1^National Institute of Chemistry, Hajdrihova 19, SI-1000 Ljubljana, Slovenia

^2^University of Maribor, Faculty of Chemistry and Chemical Technology, Laboratory for Physical Chemistry and Thermodynamics, Smetanova ulica 17, SI-2000 Maribor, Slovenia

^3^University of Primorska, Faculty of Mathematics, Natural Sciences and Information Technologies, Glagoljaška 8, SI-6000, Koper, Slovenia

-------------------------------------------------------

*Correspondence:

[milan@cmm.ki.si](mailto:milan@cmm.ki.si), National Institute of Chemistry, Hajdrihova 19, SI-1000 Ljubljana, Slovenia

[dusanka.janezic@upr.si](mailto:dusanka.janezic@upr.si), University of Primorska, Faculty of Mathematics, Natural Sciences and Information Technologies, Glagoljaška 8, SI-6000, Koper, Slovenia

**Table of contents:**

[Table S1: Important distances between atoms, for the system with 2 Mg^2+^ ions 3](#__RefHeading___Toc417299408)

[Table S2: Important distances between atoms, for the system without ions. 4](#__RefHeading___Toc417299409)

Figure S1. All-atom complex (Tm1631-DNA), protein Tm1631 and DNA RMSDs (compared to the first snapshot at 0 ns of MD) dependence against the simulation time……………………………4

Figure S2. The pKa value of Lys73 (here numbered as 72:A) and its surrounding residues calculated using the DEPTH method……………………………………………………………………………………..5

Table S1: Important distances between atoms, for the system with 2 Mg^2+^ ions. In the table are distances to Mg_1_^2+^, Mg_2_^2+^ (all 6 atoms coordinate each of the two Mg^2+^) and P (3DR) atoms. All the values are in Å.

| Atom - Distance to Mg_1_^2+^ | 1.frame | 5. frame | 7.frame | 10.frame | 15.frame | 16.frame |
| --- | --- | --- | --- | --- | --- | --- |
| TIP3 8371 OH2 | 2.03 | 1.99 | 1.98 | 1.98 | 1.96 | 1.97 |
| TIP3 12311 OH2 | 2.08 | 1.99 | 2.00 | 1.99 | 2.01 | 2.01 |
| TIP3 11859 OH2 | 2.01 | 1.94 | 1.93 | 1.93 | 1.94 | 1.94 |
| TIP3 1720 OH2 | 1.99 | 2.00 | 1.99 | 1.98 | 1.97 | 1.97 |
| TIP3 1863 OH2 | 1.81 | 2.00 | 2.04 | 2.04 | 2.05 | 2.06 |
| 3DR 7 O2P | 2.08 | 2.05 | 2.04 | 2.06 | 2.03 | 2.03 |
| **Distance to Mg_2_^2+^** |  |  |  |  |  |  |
| GLN 115 OE1 | 1.88 | 1.87 | 1.91 | 1.92 | 1.93 | 1.93 |
| CYT 6 O3' | 2.32 | 2.01 | 2.03 | 1.96 | 1.84 | 1.84 |
| ASN 45 OD1 | 1.97 | 2.09 | 2.03 | 2.10 | 2.04 | 2.05 |
| TIP3 6134 OH2 | 2.35 | 2.36 | 2.38 | 2.38 | 2.41 | 2.41 |
| 3DR 7 O2P | 2.01 | 2.06 | 2.02 | 2.06 | 2.26 | 2.32 |
| TIP3 8306 OH2 | 1.94 | 2.00 | 1.99 | 2.02 | 2.00 | 2.00 |
| **Distance to P** |  |  |  |  |  |  |
| CYT 6 O3' | 1.72 | 1.97 | 2.19 | 2.56 | 3.06 | 3.14 |
| TIP3 1863 OH2 | 2.69 | 1.85 | 1.76 | 1.73 | 1.70 | 1.70 |
| 3DR 7 O2P | 1.57 | 1.60 | 1.59 | 1.57 | 1.56 | 1.56 |
| 3DR 7 O1P | 1.48 | 1.49 | 1.49 | 1.48 | 1.47 | 1.47 |
| 3DR 7 O5' | 1.61 | 1.63 | 1.62 | 1.61 | 1.60 | 1.60 |

Table S2: Important distances between atoms, for the system without ions. All the values are in Å.

| **Atom (bond)** | **1. frame** | **4. frame** | **5. frame** | **7. frame** | **9. frame** | **10.frame** | **16.frame** |
| --- | --- | --- | --- | --- | --- | --- | --- |
| 3DR 7 O2P <-> 3DR 7 P | 1.507 | 1.507 | 1.507 | 1.512 | 1.519 | 1.517 | 1.521 |
| 3DR 7 O1P <-> 3DR 7 P | 1.497 | 1.496 | 1.498 | 1.502 | 1.506 | 1.498 | 1.499 |
| 3DR 7 O5' <-> 3DR 7 P | 1.659 | 1.655 | 1.662 | 1.691 | 1.632 | 1.644 | 1.640 |
| CYT 6 O3' <-> GLN 115 HE21 | 2.961 | 3.001 | 3.009 | 2.037 | 3.338 | 3.074 | 1.874 |
| 3DR 7 P <-> TIP3 1863 OH2 | 4.352 | 3.584 | 3.233 | 2.361 | 1.699 | 1.661 | 1.655 |
| CYT 6 O3' <-> 3DR 7 P | 1.647 | 1.656 | 1.653 | 1.849 | 2.773 | 3.074 | 4.346 |
| TIP3 6134 H1 <-> 3DR 7 O2P | 2.916 | 2.887 | 2.885 | 3.076 | 1.670 | 1.682 | 1.739 |
| TIP3 6134 H2 <-> GLN 115 OE1 | 2.227 | 2.153 | 2.162 | 2.508 | 1.672 | 1.690 | 1.681 |


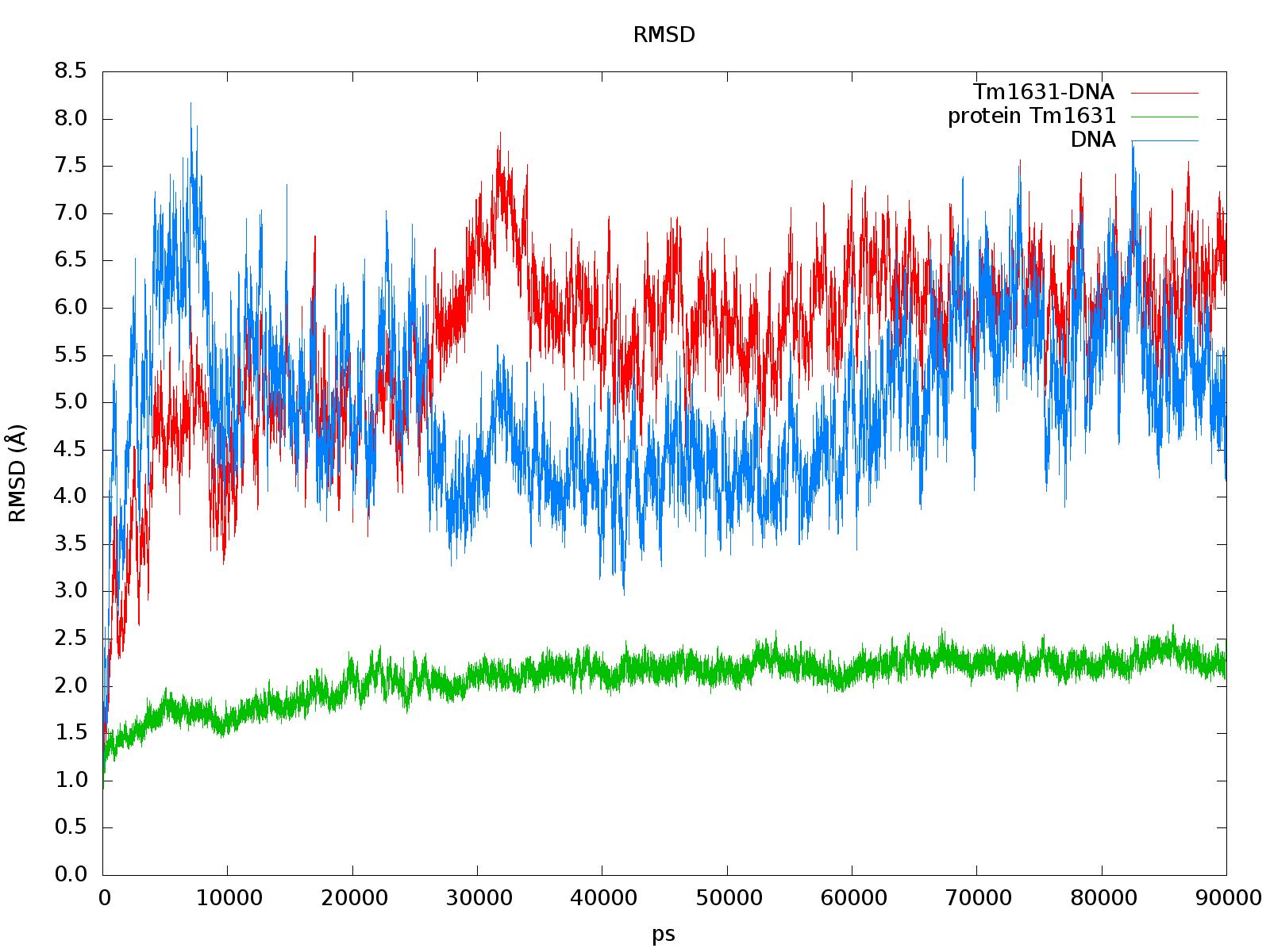


Figure S1. All-atom complex (Tm1631-DNA), protein Tm1631 and DNA RMSDs (compared to the first snapshot at 0 ns of MD) dependence against the simulation time.


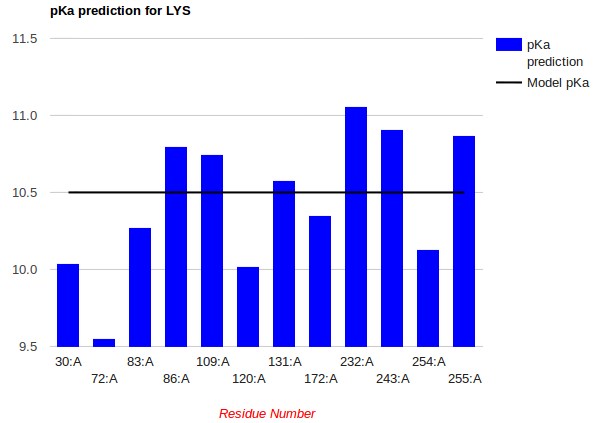


Figure S2. The pKa value of Lys73 (here numbered as 72:A) and its surrounding residues calculated using the DEPTH method.
